# Application of xCELLigence RTCA Biosensor Technology for Revealing the Profile and Window of Drug Responsiveness in Real Time

**DOI:** 10.3390/bios5020199

**Published:** 2015-04-16

**Authors:** Dan Kho, Christa MacDonald, Rebecca Johnson, Charles P. Unsworth, Simon J. O’Carroll, Elyce du Mez, Catherine E. Angel, E. Scott Graham

**Affiliations:** 1 Department of Pharmacology, University of Auckland, Auckland 1023, New Zealand; E-Mails: d.kho@auckland.ac.nz (D.K.); c.macdonald@auckland.ac.nz (C.M.); rebecca.johnson@auckland.ac.nz (R.J.); 2 Centre for Brain Research, University of Auckland, Auckland 1023, New Zealand; E-Mail: s.ocarroll@auckland.ac.nz; 3 School of Medical Sciences, University of Auckland, Auckland 1023, New Zealand; 4 Department of Engineering Science, University of Auckland, Auckland 1010, New Zealand; E-Mail: c.unsworth@auckland.ac.nz; 5 Department of Anatomy with Radiology, University of Auckland, Auckland 1023, New Zealand; 6 School of Biological Sciences, University of Auckland, Auckland 1010, New Zealand; E-Mails: elycedumez@hotmail.com (E.M.); c.angel@auckland.ac.nz (C.E.A.)

**Keywords:** xCELLigence, adhesion, cytotoxicity, cell death, drugs, responsiveness, Cell Index

## Abstract

The xCELLigence technology is a real-time cellular biosensor, which measures the net adhesion of cells to high-density gold electrode arrays printed on custom-designed E-plates. The strength of cellular adhesion is influenced by a myriad of factors that include cell type, cell viability, growth, migration, spreading and proliferation. We therefore hypothesised that xCELLigence biosensor technology would provide a valuable platform for the measurement of drug responses in a multitude of different experimental, clinical or pharmacological contexts. In this manuscript, we demonstrate how xCELLigence technology has been invaluable in the identification of (1) not only if cells respond to a particular drug, but (2) the window of drug responsiveness. The latter aspect is often left to educated guess work in classical end-point assays, whereas biosensor technology reveals the temporal profile of the response in real time, which enables both acute responses and longer term responses to be profiled within the same assay. In our experience, the xCELLigence biosensor technology is suitable for highly targeted drug assessment and also low to medium throughput drug screening, which produces high content temporal data in real time.

## 1. Introduction

This biosensor technology is designed to allow continuous real-time monitoring of the adhesion properties of cells *in vitro* in a non-invasive label-free manner. The xCELLigence system uses custom-designed plates, which have a high-density gold electrode array upon which the target cells adhere and grow. Cells adhere to the plate surface and influence the electrical impedance across the array, which is measured and recorded by the xCELLigence software. The impedance values are converted by the software into the Cell Index (CI), which is then used as a measure of adhesion (for original ACEA schematics explaining the Cell Index, see http://www.aceabio.com/theory.aspx?cateid=281). In the absence of cells, the Cell Index will be zero, and as cells adhere to the array, the Cell Index increases. In the simplest terms, the greater the Cell Index values, the greater the level of adhesion. Conversely, when the Cell Index decreases, this means that the net adhesion is decreased. In principal, xCELLigence is measuring the net cellular (focal adhesions) adhesion within the well. Therefore, any response that induces changes in cell morphology (size, volume, shape or spreading), cell number (proliferation or death) or movement (migration or extravasation) can be investigated using xCELLigence technology.

xCELLigence biosensor technology has now been validated by a range of research groups to investigate multiple complex cellular behaviours and drug responses. This includes drug effects on the viability and migration of tumour cells [1,2] and cell toxicity to drugs [3,4,5], nanoparticles [6] and immune cells [7,8]. More novel applications include using xCELLigence to screen compounds for their ability to induce adipogenesis [9] and for monitoring the differentiation of SH-SY5Y cells [10].

The development of the xCELLigence Cardio system represents a major step forward in pre-clinical drug screening to assess cardiotoxic effects, which is a common side effect of many drugs and is claimed to be a major cause of drug candidates failing in clinical testing. The xCELLigence Cardio is capable of measuring cardiomyocyte viability, whilst simultaneously measuring rhythmic beating [11,12]. This unique combination has made the xCELLigence Cardio a viable option for predicting the ability of drugs to induce arrhythmias [13].

The aim of this paper is to provide an unbiased insight into xCELLigence biosensor technology for drug response profiling applications and to explain the technology platforms and methodology required for this research. Over the past four years, we have used xCELLigence biosensor technology to: (I) optimise cell culture conditions; (II) discover drug- and cytokine-induced cell death; (III) measure immune cell-mediated target killing; (IV) as a bioassay to rapidly assess the purity of human neuronal cultures; and to (V) improve experimental design. Herein, we explain the basics of the xCELLigence biosensor and the resultant Cell Index curves. We also highlight real examples of where xCELLigence can be applied to improve cell culture techniques, experimental design, conduct toxicity studies, pharmacology and for drug screening. In our experience, the temporal profiling capacity and autonomous nature of xCELLigence are very powerful for revealing responses where little or nothing is known about the drug response and is therefore ideal for drug discovery applications.

## 2. Experimental Section

### 2.1. Cell Culture

All media, serum and antibiotics were purchased from Invitrogen (Life Technologies, Auckland, New Zealand). Cytokines were purchased from PeproTech (Rocky Hill, NJ, USA). S1P was purchased from Tocris.

### 2.2. Differentiation of Astrocytes

The NTera2/D1 (NT2) cell line was purchased from ATCC (American Tissue Culture Collection). Astrocyte cultures were differentiated form the NT2 precursors using the retinoic acid (RA) differentiation method [14,15] with various modifications. In brief, neurons were produced after a 4-week differentiation protocol using 10 µM RA [14,15], followed by 2 weeks with specific mitotic inhibitors [16,17]. Astrocytes were subsequently differentiated from the cultures after the neuronal cells had been completely removed, after a further 2–3 weeks with mitotic inhibition [18,19]. Following differentiation, astrocytes were cryopreserved in 10% DMSO, 45% FBS and 45% DMEM/F12. The standard complete media used for the NT2 astrocytes is DMEM/F12 supplemented with 10% FBS (DF10).

### 2.3. Endothelial Cultures

The HMEC-1 (human dermal microvascular endothelial cells) were purchased from the CDC (Centre for Disease Control, USA) through ATCC. Upon receipt from ATCC, the endothelial cells were expanded in T75 flasks and then cryopreserved at a low passage number (3–5). The media used for the HMEC-1 endothelial cells was MDCB-131 supplemented with 10% FBS. Human brain cerebral microvascular cells (hCMVECs) were purchased from Applied Biological Materials Inc. (Richmond, British Columbia., Canada). hCMVECs were cultured in M199 media supplemented with 10% FBS and cryopreserved at low passage (3–5).

### 2.4. Seeding Cells into xCELLigence Plates

**Figure 1:**
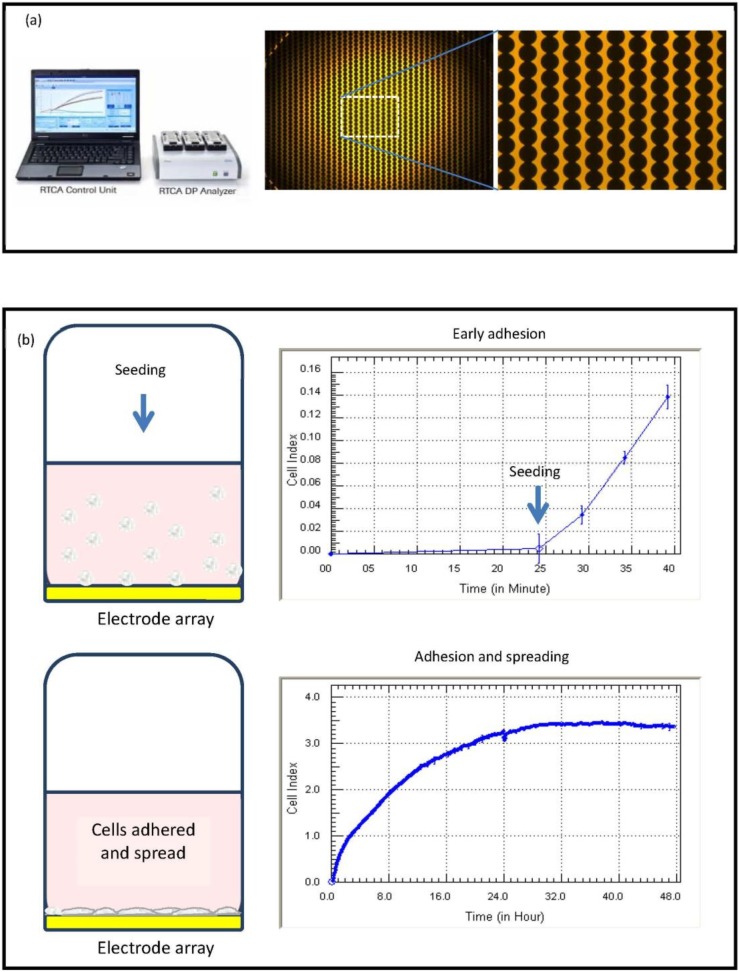
xCELLigence technology; impedance recording and cell adhesion. Summary of xCELLigence system technology and impedance, which shows: (**a**) the high density gold array on the E-plates; and (**b**) the initial phases of seeding and adhesion, which is recorded by the biosensor and represented as the Cell Index (adhesion).

The E16 or E96 xCELLigence plates were prepared by addition of complete media (50 µL) to every well. After equilibration to 37 °C, plates were inserted into the xCELLigence station, and the base-line impedance was measured to ensure that all wells and connections were working within acceptable limits. The software automatically informs the researcher if any connection problems arise. Following harvesting and counting, cells were diluted to the correct seeding density and added to the wells in 50 µL (see the schematic in Figure 1). We have found that this volume provides less variation in seeding density, especially for larger/dense cells, like astrocytes. We would recommend that researchers should seed their cells using their standard seeding protocols and assess how much seeding variation exists.

### 2.5. Using Different Growth Matrices

Certain cell types require special matrix coatings to enhance adhesion or to develop the correct cellular phenotype. Plates were coated with collagen for specific experiments. Collagen was applied to the well for 1 h, and the excess was removed and rinsed gently with sterile PBS. Care must be taken to avoid contact with the microelectrode array on the E-plate wells.

### 2.6. xCELLigence Software

**Figure A1:**
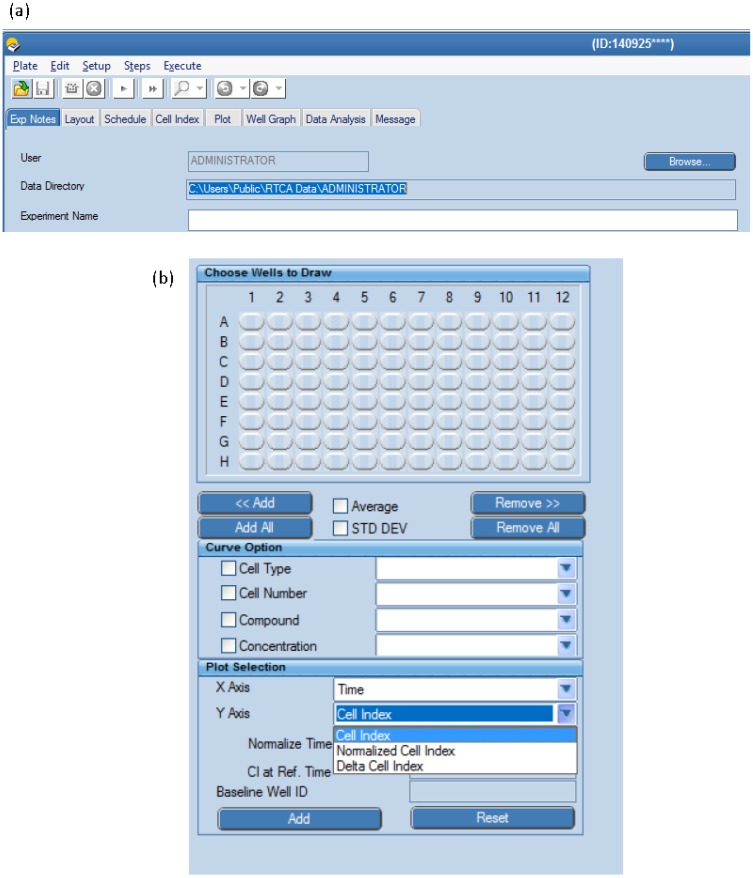
Screenshots of key software functionality. (**a**) The default software page showing the various page tabs; (**b**) highlighting how to switch between the Cell Index, Normalised Cell Index and the Delta Cell Index view in the Plot tab.

The software version used in these experiments is 1.2.1. The software is relatively easy to use and reasonable intuitive for the beginner. The software provides an electronic record of the experimental details. All experimental data in the file are permanent and cannot be altered or changed by the user, which is an excellent aspect to the design. The default open page of the current software has 8 tabs (Exp Notes, Layout, Schedule, Cell Index, Plot, Well Graph, Data Analysis and Message; see Figure A1a). The Exp Notes page is an electronic lab book. We recommend to all new users to complete this section as fully as possible. Layout is the design of the plate (e.g., cells/treatments). The Layout page is important, as the information entered here is used for the grouping of wells for calculating averages (±standard deviation) and subsequent pharmacological plots for calculating EC_50_ or IC_50_ values, which is done in the “Data Analysis” tab. The Schedule page is for the timing of the recordings. The recording interval and duration of the experiment will of course vary; but any permutation of timing is possible with the software, and emphasis should be placed on optimally recording the desired cellular response. The Plot tab is useful for visualisation of the experiment in real time. This function is extremely important as the data can be viewed as it happens, which allows xCELLigence to be run in conjunction with other end-point assays and, thus, reveal precisely when the response has occurred. In addition, the Plot tab produces publication quality graphs, and we prefer the simplicity and temporal nature of this output for most of our data. The Well Graph tab provides a snap-shot image of the entire dataset in E96 well format. The Data Analysis tab allows for more advanced pharmacology to be conducted (e.g., EC_50_/IC_50_). Finally, the Message tab alerts the user of any errors or faults and records the timing when the plate is engaged or removed from the system. Again, this record is permanent and reveals any warning signals or problems (connection issues, plate missing during sweeping), should they arise.

### 2.7. Plotting of Data

By far the easiest way to observe the data is using the Plot page. This not only displays the data in real time, but allows grouping of treatments as the mean ± standard deviation. The data can be displayed as several options: (I) as the Cell Index; or (II) the Normalised Cell Index; or (III) the Delta Cell Index (see Figure A1b). The Cell Index is the measure of adhesion across the individual well. The greatest Cell Index recording we have observed has been with astrocytes (Cell Index of 10–15, *i.e.*, very adherent cells). In the absence of cells (media only) or with suspension cells, the Cell Index values will be close to zero. It is useful to know the raw Cell Index of the cells, as this provides a measure of how adherent the cells are. The Normalised Cell Index (NCI) is a manipulation of the data, where a specific time is chosen (*i.e*., when cells were stimulated), which is then set as 1.0 (100% values) by the software. Then, all values are represented as a proportion of this. NCI is useful to estimate the percentage change in adhesion. Caution must be applied when using the NCI function, as information present in the Cell Index data is removed during manipulation to NCI. This is highlighted in Figure A3.

## 3. Results and Discussion

### 3.1. xCELLigence: Improving the Basics of Cell Culture and Experimental Design

**Figure 2:**
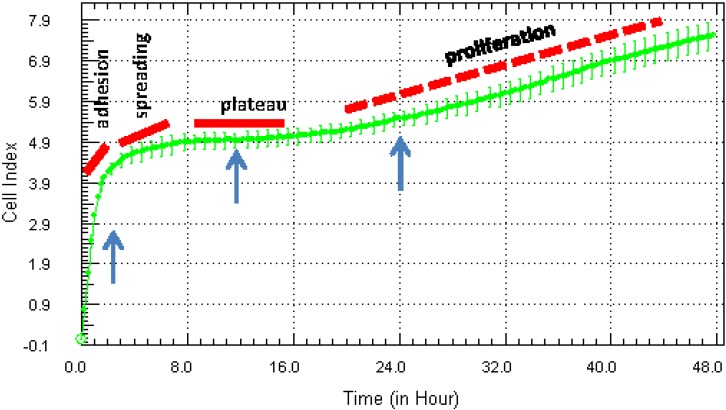
Interpretation of xCELLigence biosensor Cell Index curves: determination of assay parameters influencing experimental design. After obtaining a standard Cell Index adhesion curve for a particular cell type, it can be used to interpret specific behaviours of the cells relevant to the experimental design. Using the HMEC-1 endothelial cells as an example, the initial period of the Cell Index curve reveals the initial phase of cell adhesion and spreading (0–8 h), followed by a plateau phase prior to a gradual period of proliferation. The arrows represent potential time points for drug treatment; note that the behaviour or response of the cells may vary at each. The curve represents the mean Cell Index value from >4 wells ± SD.

Interpretation of xCELLigence Cell Index curves: The strength of cell adhesion is represented by the software as the Cell Index (unit-less measurement). As cells adhere to the E-plates, the Cell Index value increases from zero, and this will usually be evident within the first 10–15 min of seeding (see Figure 1). Cells that are very strongly adherent will reach a maximum Cell Index of ~10–15. In our experience, this is the upper range for Cell Index values. Non-adherent cells will not affect the Cell Index values greatly (0–1), whereas a Cell Index of 1–4 is weak, with 5–10 being moderate to strong. As adhesion decreases, there will be a concordant decease in the Cell Index. Figure 2 emphasizes different phases of adhesion, which occur for vascular endothelial cells (HMEC-1 cell line). These cells are strongly adherent and also proliferative until they form a monolayer. The initial increase (0–4 h) in the Cell Index is associated with the attachment and adhesion of the cells (schematics in Figure 1b and Figure 2), followed by spreading and a brief plateau phase. This is a proliferative cell line, and the progressive linear increase in the Cell Index post 24 h after seeding is consistent with the initiation of the proliferation of these cells. In theory, this temporal profile should only be observed with proliferating cells (lines), whereas non proliferating cells will remain constant until they become nutrient deprived and compromised.

Defining the window for drug treatments: In classical *in vitro* assays, treatments are often conducted at a time point that is convenient (24 h post seeding) rather than based on empirical or behavioural data from the cells. In contrast, the data provided by the xCELLigence Cell Index curves reveal extra information related to the behaviour, growth and health of the cells, which can be used to guide and improve the experimental design. This information can be used to identify the correct time window for drug treatments and to ensure that this occurs consistently across studies or experiments. In Figure 2, we have added three arrows indicating convenient time points. The aim here is to illustrate that the cells may be behaving differently at each time point, and this could influence the subsequent treatment(s).

**Figure 3:**
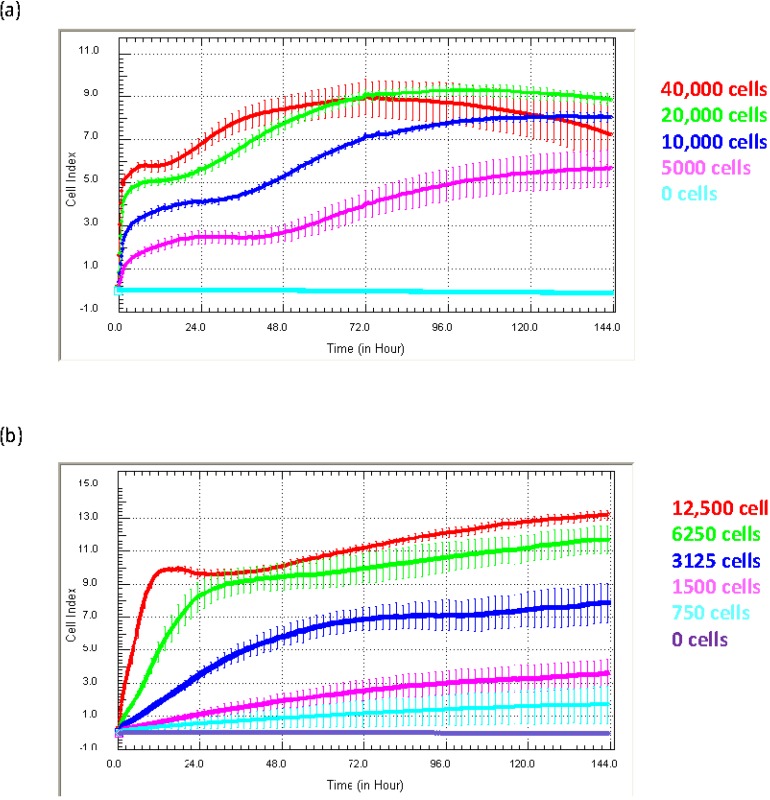
Optimisation of ideal culture conditions. Different Cell Index adhesion curves are obtained from different cells types. The adhesion of (**a**) HMEC-1 endothelial cells and (**b**) NTera2/D1 (NT2)-astrocytes is shown. Both cell types were seeded at a range of titrations to identify the ideal seeding density and reveal time windows for drug treatment. Curves represent the mean Cell Index value from >4 wells ± SD.

Defining the ideal seeding density and optimal culture conditions using the xCELLigence biosensor: Defining the appropriate seeding density and window of culture are very important aspects of the experimental design. Figure 3 shows examples of growth curves obtained from two quite different cell types, (1) endothelial HMEC-1 cells and (3) NT2-astrocytes, which have been grown at different seeding densities for six days to identify the ideal culture conditions.

Different cell types produce Cell Index curves with subtly different characteristics. The Cell Index curves reveal several important details pertaining to the kinetics of the initial adhesion (rate and strength), proliferation and survival of the cells. For the endothelial cells (Figure 3a), initial adhesion is rapid, indicated by the sharp increase in the Cell Index over the first few hours post seeding. This is followed by a period of proliferation, indicated by the progressive increase in Cell Index. This proliferative phase is observed at all seeding densities and, as expected, takes longer at lower seeding densities (Figure 3a). The Cell Index curves can also indicate when cellular compromise begins, which is marked by a gradual or progressive decline in the Cell Index. In panel (a), the endothelial cells at the highest density (40,000 cell/well) show this characteristic profile after about 72 h of culture. Being aware of this information is very important for conducting long-term studies or drug-cytotoxicity studies. Here, xCELLigence essentially removes the “best guestimate” component of experimental design, which is often used in the absence of real-time biosensor technology.

The profile of the Cell Index curves for the astrocytes (Figure 3b) is different from that of the endothelial cells. The initial increase in the Cell Index represents the initial period of astrocyte adhesion and spreading (see Figure 1). Note the substantially different time course required by the astrocytes to reach the maximum Cell Index and that at the lower densities (3125 cells and below) for which the maximum Cell Index is never reached. This is a classical observation of non-proliferating cells. Typically, the seeding titration time course reveals: (I) the ideal seeding density to use; (II) how long the cells can be cultured without compromise; and (III) it can help define when to stimulate the cells (see below). Recommendation: we strongly recommend conducting seeding titrations for all new cell types being used on xCELLigence technology. This will be advantageous when using highly valuable primary cells, where the sensitivity of xCELLigence may allow fewer cells to be used, thus providing savings of a valuable resource.

**Figure 4:**
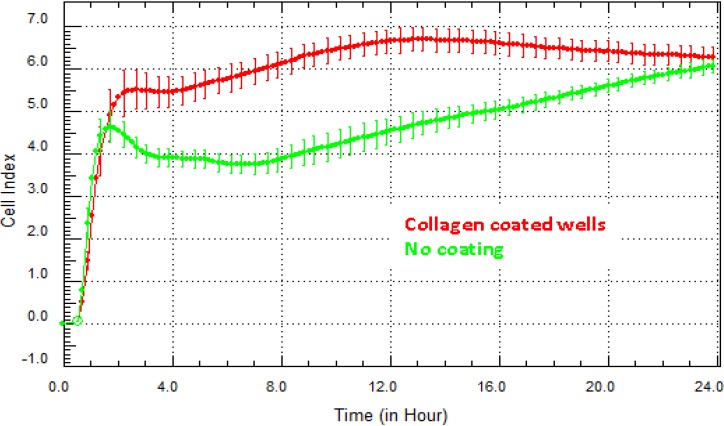
Optimisation of matrix coating for best growth conditions. There are a multitude of factors that can influence the growth and viability of cells. This figure highlights the influence of collagen (red curve) on the adhesion of human brain cerebral microvascular cell (hCMVECs) endothelial cells. Collagen is a major component of the basal lamina of the blood brain barrier structure. Here, the endothelial cells achieve a higher level of adhesion faster than those not grown on a matrix. Mean Cell Index value from >4 wells ± SD.

Optimising growth conditions using extracellular matrix coatings: Often, the conditions that produce the correct cellular phenotype or growth conditions for routine cell culture of your cells will produce the best result on xCELLigence technology, too. Figure 4 highlights the difference in the Cell Index for hCMVECs (brain endothelial cells) grown on collagen in comparison to cells grown without matrix. Collagen is an important component of the basement membrane of the blood brain barrier (BBB). It is therefore logical that collagen will improve the adhesion and growth of the brain endothelial cells. The xCELLigence biosensor demonstrates this very easily and can be used to screen which matrix coatings enhance or improve cell growth or viability. Recommendation: the SP system (Single 96-well xCELLigence Plate) provides a good option for conducting the optimisation steps in one or two experiments and can be done using a single plate. Where substantially different curves are obtained on different coatings, consideration should be given as to whether the coating affects the aspects of signaling, growth or cell behaviour relevant to the experimental outcome.

**Figure 5:**
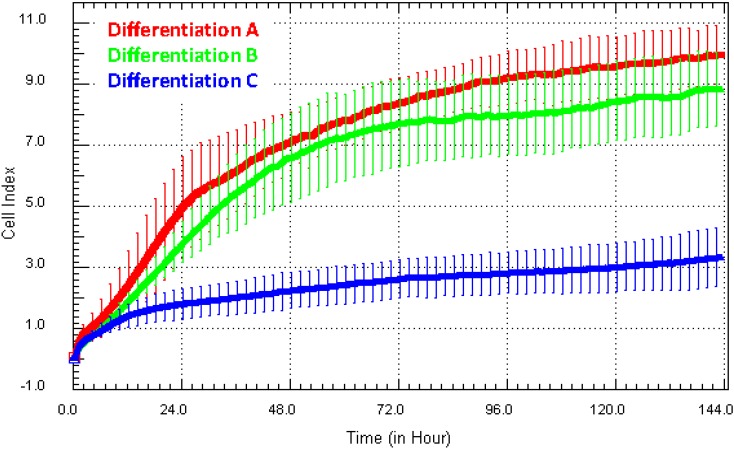
Direct comparison of growth characteristics of NT2-astrocytes from different cultures. Here, xCELLigence was used to assess the consistency and growth characteristics of NT2-derived astrocytes produced from different differentiations (and by different students). This example shows that the astrocytes in Differentiation C had a very low Cell Index, which is inconsistent with the strong level of adhesion typical of these cells (Differentiations A and B).

Comparison of variation were made between differentiations or donors (in our research, we use a range of cells derived from cell lines [5,20,21] (e.g., HEKs, HMEC-1 and hCMVECs), differentiated precursors (NT2 astrocytes [7,22,23] and neurons [24]) and from primary cells from blood [25,26] and brain [27]). The simple set-up and real-time readout of xCELLigence provides a technology to directly compare any variation in the global temporal growth characteristics of cells from different batches of differentiated cells (e.g., NT2) or from different donors. Figure 5a shows the Cell Index growth curves from three independent NT2 astrocyte differentiations. These cells are produced following an expensive 10-week differentiation protocol. Typically, we assess the success of the differentiation using GFAP/vimentin/βIII-tubulin expression to indicate the purity and yield of astrocytes. The data shown in Figure 5 clearly suggest that the growth of the astrocytes from Differentiation C (represented by the blue Cell Index curve) was substantially different from that of A and B (red and green curves). We have assessed more than 30 different astrocyte differentiations using xCELLigence, and they typically produce a Cell Index >7 (strong). The weak adhesion from Culture C suggested that the yield or health of the astrocytes was poor and therefore not suitable for further assays. Recommendation**:** in addition to comparison of differentiations (yield, quality and consistency), the long-term temporal capacity of xCELLigence is ideal for comparing the consistency of cell lines over time, especially those that may have been passaged to high numbers or genetically modified. In addition, it has applications for primary cells from different donors or the comparison between different disease states.

### 3.2. xCELLigence: Investigating Drug Responsiveness

This section provides several examples of how xCELLigence can be used for investigating drug-induced responses from cells *in vitro*. There are several points we aim to highlight, which include: (1) determining whether your cells respond; (2) determining when your cells respond; and (3) what is the nature of the response? Each of these points are important across a spectrum of pharmacological or drug discovery applications.

**Figure 6:**
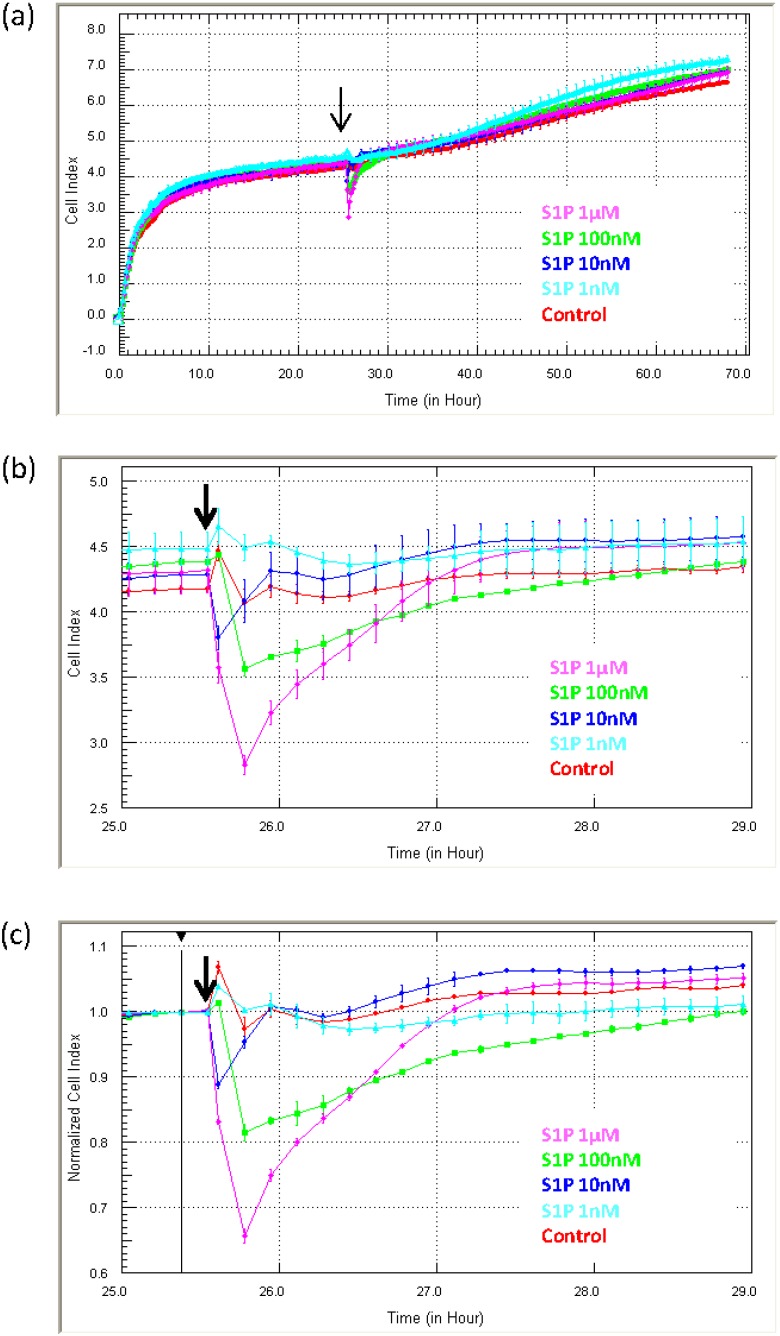
Measurement of rapid or transient cellular responses using xCELLigence. The usefulness of xCELLigence for monitoring acute transient responses is exemplified with the response of microvascular endothelial cells to sphingosine-1-phosphate (S1P). The response to S1P is immediate and transient, and xCELLigence reveals the precise timing and magnitude of the response. S1P was added at several concentrations (addition indicated by black arrow, 1 µM to 1 nM), which reveals a concentration-dependent response. The Normalised Cell Index plot is also used in conjunction with the Cell Index plots to determine the extent (%) of the response. Curves represent the Mean Cell Index value from >4 wells ± SD.

Identification of acute drug responses: Figure 6 shows the response profile of endothelial cells (HMEC-1 cells) to sphingosine-1-phosphate (S1P; 1 nM to 1 µM). The longitudinal data (three days) in Figure 6a reveal the rapid and transient reduction in adhesion induced by S1P, which occurs immediately following S1P addition (black arrow). Figure 6b focuses on the period immediately after S1P addition to highlight how fast the response occurs. Figure 6c shows the Normalised Cell Index at the time of S1P addition. It reveals that the approximate reduction in adhesion is 10%–20% within 5 min of addition and maximal (~30%) within 10 min. These data show: (I) that the endothelial cells respond to S1P across a range of concentrations; and (II) that the response is immediate and transient. The profile in (a) also demonstrate that the S1P does not induce compromise or death of the cells, which would be shown by a progressive sustained loss in the Cell Index [22]. Although xCELLigence does not reveal the biology of the response, it does reveal that there is a response and most importantly when the response occurs. We are certain that without the real-time biosensor detection, the acute immediate nature of the S1P response would have been missed. This is very useful for future experimental design to identify how S1P regulates endothelial function.

Measurement of drug-induced cytotoxicity using xCELLigence: In our experience, xCELLigence technology provides a very useful readout for measuring cell death (of adherent cells). The real-time dynamic readout provides information relating to: (I) the on-set of cellular compromise; and (II) when death is likely to be occurring. Contrast this to a single end-point assay, which reveals nothing about the dynamics of the response or the final fate.

**Figure 7:**
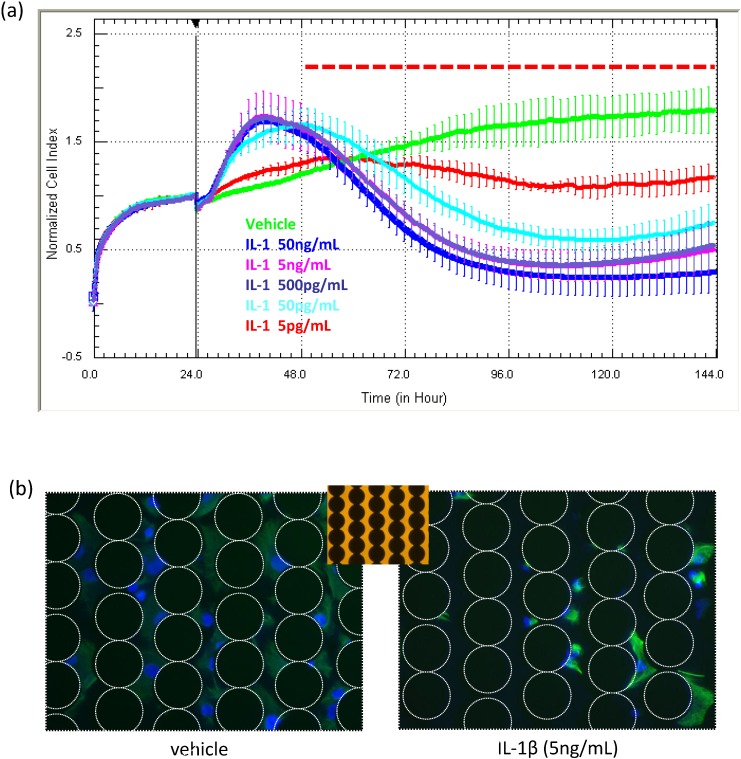
Use of xCELLigence for measuring drug effects on cell viability, compromise and death. Here, NT2 astrocytes were treated with the pro-inflammatory cytokine IL1β at a range of concentration to assess the acute and long-term effects on the cells. During the initial 24-h period following cytokine treatment, there is a pronounced increase in the Cell Index, which is consistent with inflammatory activation of the cell and increased astrocytic size as a consequence. This is followed by a progressive and continual loss of adhesion. IL1β was added 24 h after seeding, and each curve represents the mean ± SD of four wells. The images in (**b**) show the viable astrocytes remaining on the E-plate at the end of the experiment. The white dotted circles reveal the position of the non-transparent electrode array. These images were acquired using an inverted microscope. The array can be seen more easily in the bright-field insert. Note that ACEA have developed plates with view strips for imaging of cells during xCELLigence experiments.

The data in Figure 7 are unpublished data from an earlier study investigating the influence of various inflammatory cytokines on the function of NT2-derived astrocytes [22,23]. The longitudinal capacity of xCELLigence revealed that several pro-inflammatory cytokines (IL-1β and TNFα) induced compromise of the astrocytes at specific concentrations. This is highlighted in Figure 7a, exemplifying the effect of IL-1β. The initial increase in the Cell Index is consistent with the increased size and activity of the astrocytes during the initial inflammatory phase, which is soon followed by gradual progressive compromise and death. The response was concentration dependent (5 pg/mL to 50 ng/mL). In this study, the xCELLigence profile suggested that compromise was occurring 24–48 h after cytokine addition. This was confirmed using a combination of terminal assays, including cell counts, nuclear fragmentation and cleaved caspase-3 expression [22]. It is important to note that the cytokine-induced compromise of the astrocytes was only realised due to the long-term xCELLigence data. In [22], we utilized the real-time readout of xCELLigence as a precise guidance system to conduct terminal assays to determine the extent of compromise and death with conventional apoptotic assays [22] and cell counts (Figure 7b).

**Figure 8:**
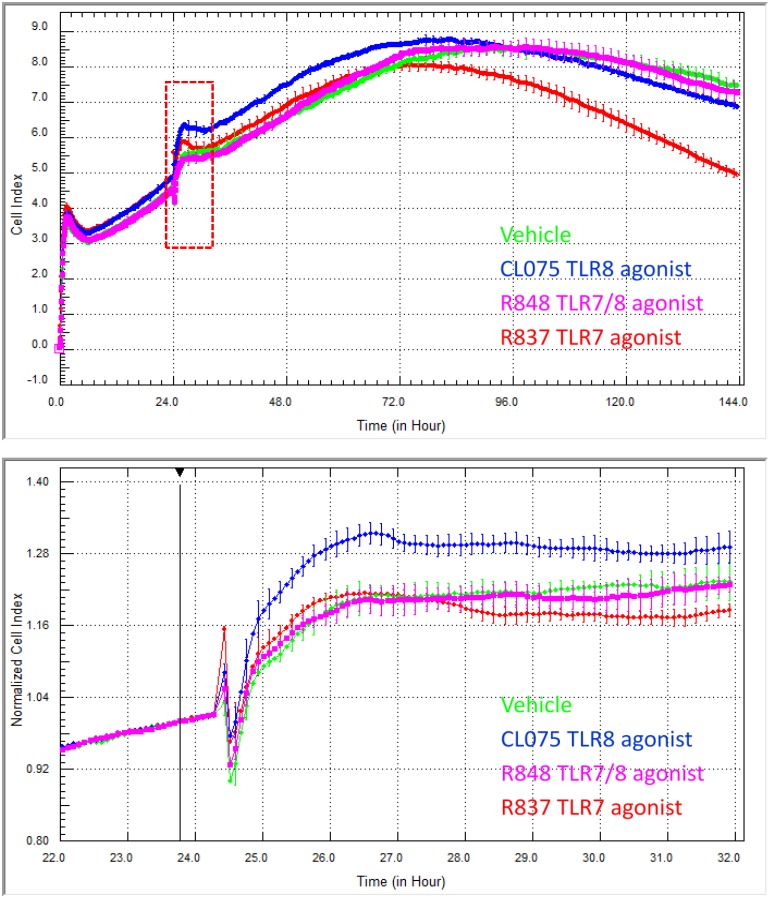
xCELLigence reveals the differential temporal responsiveness of skin endothelial cells to various TLR ligands across a six-day time course. CL075 (5 μg/mL) induces an immediate and sustained increase in endothelial adhesion, whereas the R837 (5 μg/mL) effects are not obvious until at least 48 h after drug addition. The cells did not appear to respond to R848 (10 μg/mL). The black arrow indicates the time of TLR agonist addition, and the acute response period, highlighted by the red box, is shown in the lower panel. The lower panel reveals clearly the immediacy of the CL075 response. Each curve represents the mean of four wells ± SD. Note that the lower panel is normalised to the time of drug addition.

The data in Figure 8 are a prime example of how xCELLigence can reveal drug responses that occur in very different time frames. In this study, a specific panel of TLR ligands was assessed using xCELLigence for those that regulated skin endothelial cells. We had no prior knowledge of whether the skin endothelial cells would respond, as the majority of TLR work is conducted with immune cells from blood. This select panel comprised CL075, R848 (Gardiquimod) and R837 (Imiquimod). CL075 is a thiazoloquinolone derivative that stimulates TLR8 and, at a higher concentration, can activate TLR7 in human peripheral blood mononuclear cells and in these cells is known to activate NF-κB, resulting in the production of pro-inflammatory mediators, including TNF-α and IL-12 [28]. The imidazoquinoline compound R848 (Resiquimod) is also an agonist for TLR7 and TLR8. R837 is an FDA-approved drug (active ingredient in Aldara and Zyclara) and prescription secondary medication for various skin cancers and also indicated for warts. Pharmacologically, R837 targets TLR7 and is thought to mediate its clinical effects through the activation of skin antigen-presenting cells. The goal of our experiments was to assess whether skin endothelial cells (HMEC-1 cells) responded to any of the TLR ligands in the panel and to ascertain the window of drug responsiveness (if any).

CL075 stimulated the endothelial cells immediately, resulting in a sustained increase in adhesion. The immediacy of the response to CL075 is evident in the zoomed lower panel, whereas the sustained effect is observed in the longer time frame (Figure 8). Again, the temporal nature of xCELLigence eloquently reveals this response. As the R848 is an agonist at both TLR7 and TLR8, we anticipated that it would likely induce a response, too. However, over the entire 144 h time course, there was no indication from the endothelial Cell Index curves of either an acute or long-term response (Figure 8). In contrast, the response profile to R837 represents a profile that we have rarely ever observed. That is a response profile where there is essentially no change in cellular adhesion initially (*i.e.*, the R837 Cell Index is identical to the vehicle control) over the acute response period. Then, however, there is a marked loss in endothelial adhesion, which is evident ~48 h after the R837 was added to the cells. This is an unusual type of response, which highlights the potential of this biosensor technology to pick up responses that may be missed by other assays.

Therefore, from this temporal comparison, it is possible to state that: (I) the skin HMEC-1 endothelial cells respond to both R837 and CL075; (II) the nature of the response is not the same, where the effects of CL075 are evident immediately, and this suggest that future experiments focus on this acute period following agonist addition; and (II) the effects of R837 on overall morphology and adhesion are only obvious after ~48 h. This kind of temporal information is essential for the design of downstream functional assays (such as cytokine secretion or cell compromise). This knowledge is imperative where a responsive may occur very acutely or transiently.

xCELLigence for drugs screening xCELLigence technology has a range of applications in the field of drug screening. Realised applications include: (I) global screening [22]; (II) targeted screening for a specific response (cytotoxicity [11,12]); or (III) drug pharmacology to identify specific receptor agonists or antagonists [29]. In the context of a global response, the Cell Index curve for the drug treatment would be interrogated for any difference in comparison to the control/vehicle-treated cells, the primary outcome of which would be to ascertain whether the cells respond or not.

**Figure 9:**
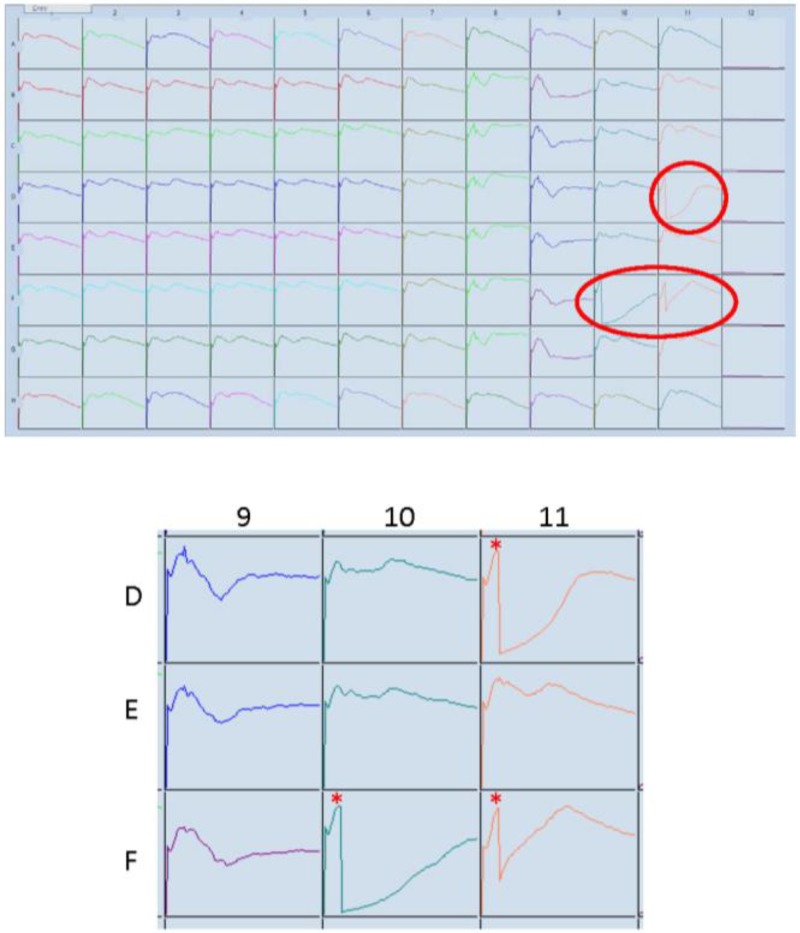
xCELLigence SP “Well Graph” view for drug discovery. An additional component of the ACEA software is the Well Graph view, which provides an overview of the entire time course for each well. This is useful for drug discovery applications or for experimental quality control. This snap-shot allows easy identification of specific or unusual responses, as highlighted by the encircled responses. These have been enlarged with the time of drug addition marked with the asterisk (24 h post seeding). The response in these highlighted wells is immediate and reveals substantial loss of adhesion, which would be consistent with a cytotoxic drug effect.

In our experience, xCELLigence provides a valuable platform to screen small compound libraries for either global responses (e.g., any change in cell adhesion) or identification of specific drug responses (e.g., cytotoxic effects). We have done this for a range of inflammatory cytokines, chemokines and various novel compounds using brain endothelial cells (hCMVECs) and NT2-derived astrocytes [22]. Figure 9 shows data where several compounds of interests induced marked death of hCMVEC endothelial cells. These responses are circled red in the Well Graph view plot (a) and enlarged in (b), whereas the control treated cells are in Column 1. The asterisk indicates the addition of compounds at 24 h post seeding. The time course of this experiment was 192 h (8 days). Several of the compounds induced immediate and substantial cell death, indicated by the rapid loss in the Cell Index. The data also suggest that the death/compromise was partial, as the initial substantial loss of adhesion is followed by a slow gradual increase in the Cell Index, consistent with proliferation of the surviving endothelial cells.

In addition, xCELLigence is an emerging platform to screen for compounds that cause cell death in the context of: (I) targeted killing of tumour cells [30]; or (II) for side-effects resulting in cardiotoxicity [11,12]. These are not applications that we have experience with specifically. However, this is a growing area in the xCELLigence RTCA literature. Moreover, cardiotoxicity is a major cause of drugs failing in clinical trials. This resulted in ACEA developing the unique RTCA Cardio system, which can measure both cardiomyocyte viability and beating simultaneously (see http://www.aceabio.com).

Recommendation: The SP (96 well) machine provides a good system for low to medium throughput. However, for higher throughput drug screening, the high-throughput (RTCA xCELLigence HT) systems (1 × 384 well) or the MP system are available (Multi-Plate 6 × 96 capacity) options.

### 3.3. Supplemental Applications

**Figure A2:**
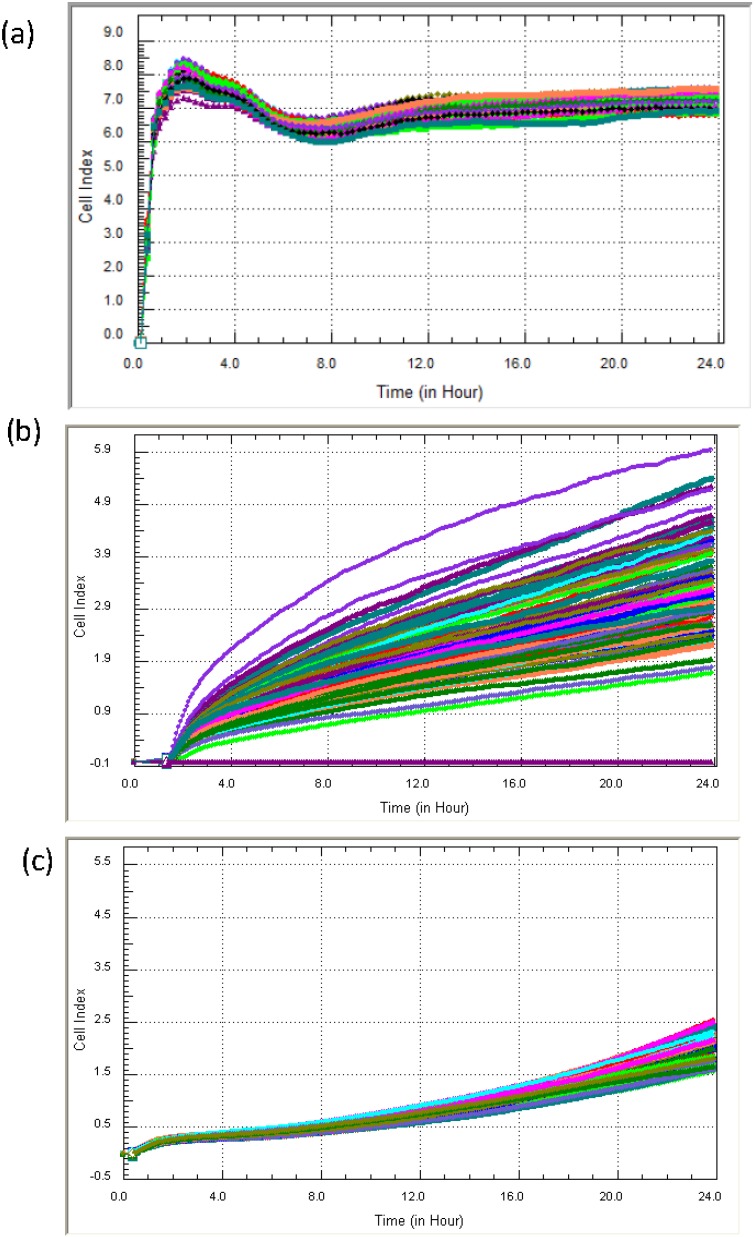
xCELLigence provides quality control and assay variation. The Cell Index curves in (**a**) show the normal variation that we observe across an E96 well plate, which is usually around 10%–15%. However, xCELLigence can also reveal poor seeding practice (**b**), which is indicative of such a substantial spread in the Cell Index curves. (**c**) An example where too few cells were seeded into the E-plate. These exemplify useful teaching and quality control aspects of the technology.

Revealing good cell culture practice: The real-time measurement of net adhesion in every well provides a valuable quality control measure for cell culture technique. In Figure A2, three very different plots are shown; Figure A2a is an example of the normal variation to expect from good cell culture practice; Figure A2b inconsistent seeding density due to poor pipetting technique; and Figure A2c poor technique in terms of being able to count cells. Each of these plots show the variation in seeding across E96 well plates with each curve representing a separate well. We have noted that large/dense cells, such as astrocytes, can sediment during seeding, which can result in substantial variation in seeding density. xCELLigence reveals this easily and therefore provides an internal quality control. We typically observe 10%–15% variation in the Cell Index across all wells of an E96 well plate, as shown in Figure A2a. However, on occasions and in particular with new practitioners of cell culture, a poor seeding technique Figure A2b or counting technique can be revealed using xCELLigence. This can be an extremely valuable teaching tool for new students. Recommendation: if the level of variation shown in Figure A2b,c is observed with an xCELLigence assay, it is likely to be occurring in other aspects of the cell culture, too!

**Figure A3:**
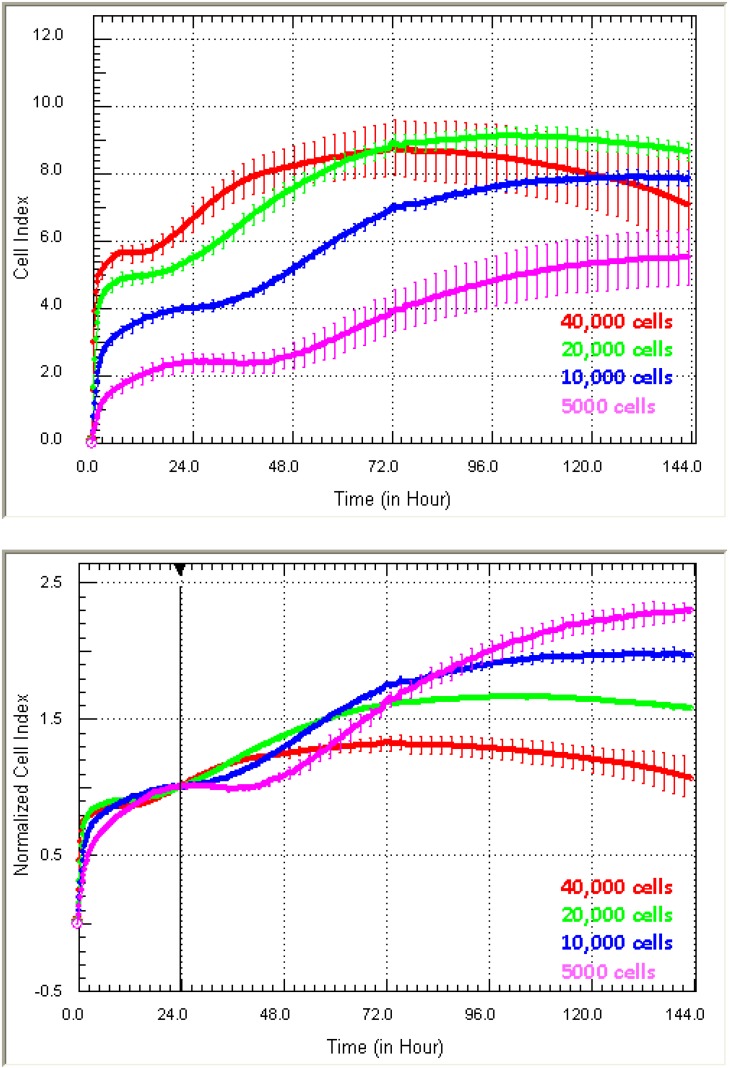
Cell Index *vs.* Normalised Cell Index. (**a**,**b**) show exactly the same dataset, where different densities of endothelial cell were grown for 144 h. The top panel shows the raw Cell Index data, whereas the bottom panel is normalised at 24 h (used here as a hypothetical drug treatment time point). Note the very different shapes of the Cell Index curves following normalisation. As the normalisation function converts all values to a proportion of 1.0, important information is lost in the transformation.

Cell Index or Normalised Cell Index: The two main ways that xCELLigence data are presented in publications is as the Cell Index or Normalised Cell Index. The normalisation function is used after a specific time point is selected (e.g., prior to drug treatment). The Cell Index values at this time are converted to 1.0, and then, every value thereafter is represented as a proportion of 1.0 relative to the change in the Cell Index. Thus, an increase from 1.0 to 1.5 would indicate a 50% increase in the Cell Index. It is our preference to use the Cell Index, which we have used throughout this paper, rather than the Normalised Cell Index for several important reasons. The Cell Index is essentially the raw data and shows the real level of variation across the experiment. In a poorly-seeded or controlled experiment, this variation can be hidden using the normalisation function. Furthermore, with the Cell Index, the consistency of the cellular adhesion achieved is retained. For example, if some experiments within the same study have a Cell Index of 6–7, whereas others achieve 12–13, then there are clear difference in the experimental setup, preparation of the cells or seeding. This information is lost when the data are normalised to 1.0. Furthermore, for work conducted in different labs, it is important to know whether researchers are achieving the same level of Cell Index from similar primary cells or cell lines. This information is only present in the raw Cell Index, which will be on a scale of 0–15 (approximately). Figure A3 highlights these points: (a) shows astrocytes seeded at different densities and the resultant Cell Index values obtained as a function of time; (b) is the same data normalised at 24 h, and this highlights how easily the normalisation function can artificially hide variation that may exist in the initial adhesion period. Note also how the magnitude of the raw Cell Index values is loss. Therefore, there is no way of knowing whether these cells were weakly adherent (Cell Index of 1–3) or extremely adherent (Cell Index of 10–15). This type of variation admittedly should never be normalised in the first place; however, unless reviewers or authors are presented with the raw Cell Index data, it would not be that easy to tell whether the normalisation was justified. Recommendation: we strongly recommend that where normalisation is used that the raw Cell Index data are also provided. The software annotates the plots to specify them as the Cell Index or Normalised Cell Index; therefore, it is easy to establish which function is used.

Advantages, disadvantages and concluding remarks: It is now evident that xCELLigence biosensor technology has numerous applications for basic research and for clinical drug development. These range from the basics of good cell culture techniques to more advanced applications, including high throughput drug screening [31] (e.g., cardiotoxicity [32,33]), cell mediated killing [7], toxicology [34] and immunology [8,35]). As our experience grows, we realise more and more applications where xCELLigence technology is valuable. The purpose of this paper is to share our experience to highlight applications, especially for researchers currently using classical end-point assays. Undoubtedly, the clinical applications for xCELLigence will continue to grow, as it represents a powerful platform for screening drug toxicity using human cells [32,33].

System considerations: Currently, there are a number of different systems that comprise the xCELLigence family. All of the research presented here has been conducted using the SP model (single E96-well platform) or the DP model (Dual-Plate; three independent E16-well plate platform). The DP model is also compatible with the migration plates (E16 cell invasion and migration (CIM) plates) for conducting tissue invasion studies. In addition to these platforms, there is also the MP model (6 × E96 well plates), the high throughput E384 well HT-system and the Cardio platform. There are several important considerations for the acquisition of a particular model, including: (1) budget; and (2) applications of the system. For the budget, the on-going costs of E-plates should be considered, in addition to the initial capital cost of the system and any institutional depreciation charges (if applicable). For applications, the main consideration is experimental scale: E16 *vs.* E96 *vs.* E384 *vs.* 6 × E96 wells. The DP system offers the opportunity to conduct three independent small-scale experiments simultaneously and is the only platform compatible with the invasion CIM plates. In our experience, it is particularly useful to have the lower throughput DP system with multiple analysis stages and the SP system for larger medium throughput studies. An impressive addition to the xCELLigence suite has been the xCELLigence Cardio platform, which encompasses dual measurement of the Cell Index and beating of cardiomyocytes in real time for the assessment of drug toxicity or effects on arrhythmias [11,12,13,32,33,36]. In addition, the RTCA-HT (high throughput; 1 × E384 well plate) and MP (6 × E96 plates) provide solutions for conducting HTS drug screening.

Advantages of the technology: We have identified and highlighted throughout this paper a number of advantages. These include: (I) the real-time or temporal nature of the data; (II) the measurement is label-free and non-invasive; (III) the impedance measurement is only affected by living adherent cells; (IV) the application of the technology to many different types of response; and (V) high throughput capacity and high content data.

Observing responses in a temporal manner is very powerful and more meaningful than end-point assays. This is exemplified by the S1P endothelial experiments, where the xCELLigence data revealed the precise period of the response and the appropriate window for further functional investigations. xCELLigence enables short- and long-term responses to be investigated in the same well, which represents a substantial savings in cells in comparison to classical end-point experiments. In addition, the availability of the data in “real time” is also very powerful. This is particularly relevant when dealing with human primary cells, where the onset or duration of the response may vary. In this scenario, xCELLigence can be used in parallel with other functional assays as a guidance system to reveal exactly when the response is occurring (e.g., collection of samples for proteomic or microarray analysis).

Disadvantages/limitations of the technology: With any new technology, there will be limitations to its use. For some researchers, the initial capital cost of the system and on-going consumables cost of the customised E-plates will be prohibitive. ACEA have recently introduced cheaper PET-bottomed plates (polyethylene terephthalate), which are an alternative to the more expensive glass bottom plates originally released by ACEA. Recommendation: the potential of xCELLigence is unlikely to be fully realised until it is used directly. Therefore, we would recommend to new users to obtain an instrument demonstration and obtain first-hand experience.

An obvious limitation is for the direct measurement of responses from non-adherent [7] or poorly adherent cells, such as neurons [24]. That said, we have used xCELLigence to measure the killing activity of human NK cells, where the death of the target cells was measured directly [7]. However, the use of xCELLigence to measure the death or proliferation of suspension cells would not be possible directly.

As xCELLigence measures net adhesion and as adhesion can be affected by a large number of cellular responses, it is not always obvious to the new user or paper reviewer what the xCELLigence data are showing. This limitation can be addressed in several ways. Where a definitive conclusion is stated from a Cell Index response curve, for example “cell death”, then this should be supported with complementary data that corroborate the change in cell number or show the mechanism of death (or both). The temporal nature of the xCELLigence data is strongly suggestive of a response, but does not prove it without the appropriate validation or supporting data. In certain situations, xCELLigence is used simply to confirm that a response has occurred or to show when it has occurred. Therefore, in this scenario, the xCELLigence data provide the supporting evidence to enable additional conclusive experiments to be conducted.

Several years ago, ACEA developed E-plates more suitable for microscopy imaging post xCELLigence acquisition. These are suitable for imaging, as a portion of the well does not contain the electrode array. To the best of our knowledge, it is not practical to combine conventional imaging simultaneously with the xCELLigence recordings. Potentially, this could only be done using an upright live cell imaging system (CO_2_ and temperature controlled) with a long working distance (above the wells), but the microscope stage would have to be oversized to accommodate the xCELLigence station.

All new technologies will experience a lag period before becoming mainstream or until the wider research community is sufficiently familiar. Thus, for all new xCELLigence applications, there is the requirement and demand to fully and exhaustively validate the xCELLigence data and to prove that the interpretation is correct. This should not be considered a disadvantage, but rather good rigorous science.

## 4. Concluding Remarks

It has been almost a decade since ACEA released its first impedance biosensor platform and six years since the launch of xCELLigence. Like any technology, xCELLigence has limitations, but these are almost insignificant to its potential. We believe that xCELLigence represents a new frontier for the study of cell biology *in vitro*, both for the advancement of basic science and for future clinical applications.
